# A service evaluation of phased- and stepped-care psychological support for health and social care workers during the COVID-19 pandemic

**DOI:** 10.1192/bjo.2023.66

**Published:** 2023-05-25

**Authors:** Charles L. Cole, Charlotte Barry, Rob Saunders, Jo Billings, Joshua Stott, Joshua E. J. Buckman, Talya Greene, Mirko Cirkovik, Stephen Pilling, Jon Wheatley

**Affiliations:** Research Department of Clinical, Educational, and Health Psychology, University College London, UK; CORE Data Lab, Centre for Outcomes Research and Effectiveness (CORE), Research Department of Clinical, Educational and Health Psychology, University College London, UK; and Talk Changes (City & Hackney IAPT), Homerton Healthcare Foundation Trust, UK; Research Department of Clinical, Educational, and Health Psychology, University College London, UK; and CORE Data Lab, Centre for Outcomes Research and Effectiveness (CORE), Research Department of Clinical, Educational and Health Psychology, University College London, UK; Division of Psychiatry, University College London, UK; Research Department of Clinical, Educational, and Health Psychology, University College London, UK; and ADAPT lab, Research Department of Clinical, Educational and Health Psychology, University College London, UK; Research Department of Clinical, Educational, and Health Psychology, University College London, UK; CORE Data Lab, Centre for Outcomes Research and Effectiveness (CORE), Research Department of Clinical, Educational and Health Psychology, University College London, UK; ADAPT lab, Research Department of Clinical, Educational and Health Psychology, University College London, UK; and iCope – Camden and Islington Psychological Therapies Services, Camden & Islington NHS Foundation Trust, UK; Research Department of Clinical, Educational, and Health Psychology, University College London, UK; Talk Changes (City & Hackney IAPT), Homerton Healthcare Foundation Trust, UK

**Keywords:** Primary care, common mental disorders, trauma, psychosocial interventions, cognitive–behavioural therapies

## Abstract

**Background:**

The COVID-19 pandemic has disproportionally affected the mental health of health and social care workers (HSCWs), with many experiencing symptoms of depression, anxiety and post-traumatic stress disorder. Psychological interventions have been offered via mental health services and in-house psychology teams, but their effectiveness in this context is not well documented.

**Aims:**

To evaluate a stepped-care psychological support pathway for HSCWs from Homerton Healthcare Foundation Trust in London, which offered psychological first aid, evidence-based psychological therapies and group-based well-being workshops.

**Method:**

The service evaluation used a pre–post approach to assess depression, anxiety, functional impairment and post-traumatic stress disorder symptom change for those who attended sessions of psychological first aid, low- or high-intensity cognitive–behavioural therapy or a combination of these. In addition, the acceptability of the psychological first aid sessions and well-being workshops was explored via feedback data.

**Results:**

Across all interventions, statistically significant reductions of depression (*d* = 1.33), anxiety (*d* = 1.37) and functional impairment (*d* = 0.93) were observed, and these reductions were equivalent between the interventions, as well as the demographic and occupational differences between the HSCWs (ethnicity, staff group and redeployment status). HSCWs were highly satisfied with the psychological first aid and well-being workshops.

**Conclusions:**

The evaluation supports the utility of evidence-based interventions delivered as part of a stepped-care pathway for HSCWs with common mental health problems in the context of the COVID-19 pandemic. Given the novel integration of psychological first aid within the stepped-care model as a step one intervention, replication and further testing in larger-scale studies is warranted.

The impact of the COVID-19 pandemic has largely been measured by the number of cases, hospital admissions and deaths, which have defined multiple peaks of infection, with differing rates of hospital admission and mortality (March to May 2020, October 2020 to March 2021, December 2021 to March 2022, June 2022 onward). Currently, the Omicron subvariants yield high levels of infection without the degree of severe illness observed during the first two waves that were fuelled by the original and Alpha variants.^1^ In each wave, health and social care workers (HSCWs) have faced exceptional demands and pressures when providing care to the public, especially during the peaks or ‘eye of the storm’. Within these acute phases, HSCWs have been exposed to potentially traumatic incidents and distressing work-related experiences, which can serve as risk factors for the later development of mental health problems, such as anxiety, depression and post-traumatic stress disorder (PTSD).^2^ Outside of these peaks, HSCWs have continued to encounter detriments to psychological well-being, such as burnout, grief and compassion fatigue, which reflect the so-called ‘recovery’ or ‘reconstruction’ phase of the pandemic.^3,4^ In recognition of these phases of distress, guidance has been issued that highlights the varying support needs of HSCWs, with the aim of mitigating the development of mental health problems in the face of experiences such as moral injury, or treating them following their emergence.^5–7^ Policy makers have emphasised offering support that is flexible, evidence-based (or at least evidence-informed) and sensitive to the needs of individuals that are disproportionately affected by COVID-19, such as front-line workers, redeployed staff and individuals from minority backgrounds.^8–11^

## The phased- and stepped-care model of psychological support

One of the first published protocols for support of this nature, which serves as the focus of the current evaluation, was described by Cole et al^12^ in a pathway inspired by the Ebola Psychological Support Service (EPSS) in Sierra Leone.^13^ This comprised three main components: rapid and prioritised psychological assessments, psychological first aid (PFA) to support distressed or potentially traumatised HSCWs during or shortly after the peaks, and psychological interventions (low-intensity cognitive–behavioural therapy (LICBT), high-intensity cognitive–behavioural therapy (HIT) and group well-being workshops). Taken together, the support offered was both ‘phased’ and ‘stepped’ (i.e. ‘the least intrusive, most effective intervention first’),^14^ with PFA provided as a novel feature of step 1. To accommodate for this, the workforce and resources were reallocated from an Improving Access to Psychological Therapies (IAPT) service. By drawing on a tried and tested model (EPSS), existing interventions and IAPT resources, the pathway was able to rapidly accept referrals without ‘reinventing the wheel’. Although psychological support is routinely offered by employment assistance programmes and other care providers to HSCWs across the UK, there are currently no published evaluations of the interventions offered by primary care (IAPT) services in particular.^15^ However, some studies have sought to understand the experience of staff accessing and delivering interventions by using qualitative research methods.^16,17^

## Aims

The primary aim was to evaluate the novel psychological support pathway provided within an existing IAPT service, established specifically for HSCWs during the pandemic.^12^ Given that some HSCWs were expected to be more adversely affected by the pandemic than others, a second aim sought to explore whether effectiveness was associated with redeployment status, ethnicity and occupation. The final aim was to determine the acceptability of the interventions offered by the service.

## Method

### Participants and setting

HSCWs of any role were eligible for support if they worked for the Homerton Healthcare NHS Foundation Trust (HHFT) in an acute or community service, or worked for another Trust but were under the care of a general practitioner in the London Borough of Hackney or the City of London. The support offered to HSCWs was in addition to the support already being offered internally by the Trust (e.g. reflective spaces and peer support). HSCWs were able to self-refer via a dedicated portal (www.talkcovidhuh.com) or through the local IAPT service's website. The care pathway was promoted in the following ways: Trust webinars, adverts via Trust communications, screensavers, testimonials, leaflets, signposting by managers, outreach workshops and word of mouth. Upon referring, the needs of HSCWs were assessed with priority (within 2 weeks of the referral date).

### Interventions

The interventions offered to HSCWs had two main aims: (a) to mitigate and prevent the emergence of mental health problems linked to potentially traumatising and/or highly distressing workplace experiences and (b) to treat common mental health problems that emerged since, or were exacerbated by, the onset of the COVID-19 pandemic. Interventions were provided by a dedicated team of IAPT staff, including psychological well-being practitioners and high-intensity therapists, all of whom were reassigned to work on the pathway as their Trust employment predated the start of the pandemic. Trainee clinical psychologists also delivered the interventions as part of a placement. All practitioners received training in adapting the interventions for HSCWs, attended case management supervision and had access to specialist group supervision. Therefore, the interventions offered were more bespoke than those offered routinely to the general public (i.e. ‘treatment as usual’). Because of the risk of infection and social distancing measures, the majority of intervention sessions were offered remotely (i.e. telephone or video call platforms); a trend observed during the initial peaks of the COVID-19 pandemic.^18^

#### PFA

PFA is an ‘evidence-informed’ approach to alleviating distress, defined as ‘humane, supportive and practical help to fellow human beings suffering serious crisis events’.^19^ It has been used to support people and communities exposed to a wide range of traumas, inclusive of those on a societal or global level. Although it is commonly used by humanitarian response workers under the guidance of the World Health Organization and North Atlantic Treaty Organization, its quantitative evidence base for efficacy is limited.^20,21^ Nevertheless, it is generally considered to be helpful rather than harmful, and has been shown to adequately prepare individuals to support others in acute distress.^22^ The UK Government has provided PFA training to the general public and healthcare professionals in response to the COVID-19 pandemic, focused on the following components of PFA: psychoeducation, comfort, protection from immediate threats to safety, practical support, provision of coping strategies, fostering social connection and information sharing.^23^

In line with the first aim of the service pathway, PFA was offered to HSCWs who were exposed to potentially traumatising or highly distressing COVID-19-related experiences in the workplace; for example, heightened concerns about the safety of themselves or close others, practical and environmental issues such as high workloads with limited support or growing patient mortality, abuse or violence from members of the public and morally injurious decisions.^5^ As these experiences were more common during the acute phases of the pandemic (i.e. high cases of infection, hospital admission and death), PFA was the first-line intervention offered under these ‘eye of the storm’ circumstances. Given this, PFA was considered a ‘step 1’ intervention as part of the overall support pathway because of its aim of alleviating acute trauma reactions and symptoms of mental health problems in their earliest form, before an intervention of greater intensity was required (i.e. psychological therapy).^14^

To protocolise the delivery of PFA, a guide was developed, which is available online.^25^ This guide describes the main components of PFA (comfort, normalise, educate, connect, provide, safety and protect), which are brought together for the recipient as a ‘well-being plan’ (including coping strategies) that is implemented between sessions to manage distress and promote well-being.^24^ The guide also prompted the practitioner to explore the recipient's social identity, which may or may not have moderated their experience of distress. As noted in the guide, two to four sessions of 30–60 min in length were recommended, which could be offered to HSCWs when they were at work, on a break or at home. To aid clinical decision-making regarding PFA eligibility, criteria were developed by members of the Covid Trauma Response Working Group (C.L.C., J.B. and T.G.) and included in the guide. Most importantly, these criteria highlight the importance of using PFA sessions to actively monitor HSCW mental health for 4 weeks after trauma exposure, rather than offering immediate psychological intervention of higher intensity, since ‘intervening in people's natural coping mechanisms too early can be detrimental’.^5^ A copy of the guide can be found in the reference list.^25^

#### Low- and high-intensity psychological therapies

HSCWs were offered the psychological interventions of LICBT or HIT for depression, anxiety disorders and PTSD, as per national treatment guidelines. These were delivered in accordance with standards specified by the IAPT manual and formed steps 2 and 3 of the pathway.^26–30^ HSCWs could access these therapies irrespective of whether they had PFA or attended any group intervention. To encourage uptake, a ‘screen and treat’ initiative was implemented across the HHFT, with mental health practitioners offering ‘drop-ins’ where HSCWs could be signposted to the service when appropriate.

#### Well-being workshops

These group interventions served the following functions: to encourage referrals for one-to-one support, to provide psychoeducational coping strategies and to foster systemic resilience by drawing on team processes. When requested by managers, the workshop facilitator(s) met with team leaders across the hospital and community settings to ascertain the main issues that HSCWs were facing, which then informed the content of the workshops that would last around 1.5 h, remotely (on Zoom) or in person. Because of the pre-workshop consultation process, these workshops became known as ‘bespoke’ workshops within the Trust, although the following content was regularly covered: ‘switching off’ (attention focus training), boosting mood (activity scheduling), unhelpful coping, managing challenging emotions, anxiety and worry, and moral injury. Also embedded within these workshops were ‘20 min care spaces’, which were designed to elicit self- and team compassion among attendees, as well as raise awareness of the challenges they faced individually and collectively.^31^ Although attendance was not compulsory, HSCWs were encouraged to attend and given protected time to do so.

### Outcome measures and data collection

Data included the IAPT minimum data-set, comprising the Patient Health Questionnaire-9 (PHQ-9^32^), Generalised Anxiety Disorder-7 (GAD-7^33^) and Work and Social Adjustment Scale (WSAS^34^), as instructed by the IAPT manual.^30^ They were collected session by session for HIT and LICBT, but were only collected pre- and post-PFA. For those who received PFA, the Global Rating of Improvement (GRI) scale^35^ was used to capture subjective changes in well-being. In addition to these measures, the self-referral portal asked whether HSCWs had been exposed to ‘potentially traumatising events at work’. Those answering ‘yes’ were invited to complete the Traumatic Screening Questionnaire (TSQ)^36^ to support decisions of whether HIT for PTSD was required. All measures completed were considered at two time points for later analysis: pre-intervention (assessment) and post-intervention (the final session).

The redeployment status (assignment of staff to a new temporary role as a result of service restructuring in face of COVID-19 pressures) and staff group/role of HSCWs were also collected, along with demographic variables (age, gender, ethnicity) routinely recorded in IAPT. All data were stored on the PCMIS case management system and anonymised at the point of extraction for analyses. Unless people ‘opt-out’, data can be used for service evaluation or improvement purposes, with consent provided at the point of self-referral. Since the GRI and TSQ measures are not routinely collected by IAPT services, permissions from PCMIS were sought to have these added to the system to support this evaluation, thereby extending the consensual agreement to include them.

To provide insight into the acceptability of PFA and the well-being workshops, two feedback questionnaires were designed (Supplementary Material available at https://doi.org/10.1192/bjo.2023.66). These questionnaires were distributed to teams after they had attended a workshop or as part of a review session with a practitioner (C.B.) who did not provide the PFA sessions, to minimise bias. These questionnaires explored HSCW satisfaction with the varying components of the interventions. Open-ended questions were also included to gather qualitative data to shape and improve the interventions being offered.

### Design and analysis

The evaluation uses a naturalistic, pre–post design to explore effectiveness of the interventions (PFA, LICBT and HIT; alone or in combination) as part of a service evaluation. Analyses were performed with SPSS for Windows, version 27.0.^37^ Paired sample *t*-tests were conducted to compare mean symptom measure scores pre- and post-intervention, and effect sizes (*d*) were calculated. Linear regression models, with the post-intervention score on each measure as the outcome variable, were constructed to determine whether ethnicity, staff group, redeployment status or intervention type(s) was associated with end-point symptom scores, controlling for pre-intervention scores. Descriptive statistics and frequencies were calculated for demographic variables and the feedback data for PFA and the well-being workshops.

### Ethical approvals

National Health Service ethical approval was not required for this paper. The data were provided by the IAPT service for evaluation as part of a wider service improvement project conducted in accordance with the procedures of the host institution and the NHS Trust that operates the IAPT service.

## Results

**Fig. 1:**
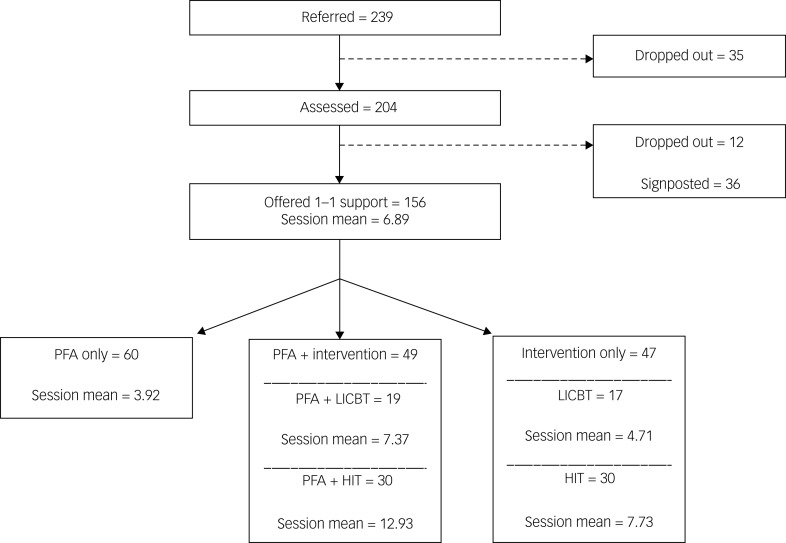
Participant flow throughout the study. ‘Session mean’ indicates the mean number of sessions attended. HIT, high-intensity cognitive–behavioural therapy; LICBT, low-intensity cognitive–behavioural therapy; PFA, psychological first aid.

A small number of cases were not included in the evaluation as they did not provide data at time point 2 (Fig. 1). Demographic data of HSCWs self-referring for one-to-one support (*n* = 239) are presented in Table 1. Although a register was provided for well-being workshops, not enough were completed to provide sufficient demographic data of those who attended. Fifty-two workshops were delivered, with between four and 16 attendees per group. It is estimated that over 300 HSCWs attended a workshop. Taken together, up to 459 HSCWs received a form of support, although this estimate may include staff who attended both one-to-one sessions and a well-being workshop.

**Table 1:** Demographics and characteristics of health and social care worker referral and intervention samples

Demographic/characteristic	Referrals (*n* = 239)		Intervention sample (*n* = 156)		*χ*^2^ *P*-values
	*n*	%	*n*	%	
Gender					
Male	38	16%	23	15%	0.756
Female	201	84%	133	85%	
Ethnicity					
White	145	61%	99	64%	0.734
Black/Black British	40	17%	23	15%	
Asian/Asian British	22	9%	12	8%	
Mixed	13	5%	12	8%	
Other	4	2%	4	3%	
Not specified	15	6%	6	4%	
Staff group					
Allied health professionals	64	27%	52	33%	0.582
Administrative and clerical	26	15%	15	10%	
Nursing and midwifery	73	31%	52	33%	
Additional clinical services	26	11%	12	8%	
Medical and dental	21	9%	13	8%	
Estates and ancillary	7	3%	5	3%	
Students	2	1%	0	0%	
Professional scientific and technical	2	1%	2	1%	
Not specified	8	3%	5	3%	
Redeployment status					
No	148	62%	87	56%	0.398
Yes	79	33%	62	40%	
Not specified	12	5%	7	6%	
Measures	Mean (s.d.)	*n*	Mean (s.d.)	*n*	*t*-Test *P-*values
PHQ-9	11.90 (5.27)	227	12.28 (5.20)	156	0.486
GAD-7	11.74 (5.17)	226	11.79 (5.05)	155	0.926
WSAS	17.29 (8.23)	215	17.68 (8.47)	149	0.661
TSQ	5.15 (2.90)	171	5.08 (2.97)	127	0.839

Eighty-three workers were not offered a form of support because of drop-out or signposting to a more suitable service. PHQ-9, Patient Health Questionnaire-9; GAD-7, Generalised Anxiety Disorder-7; WSAS, Work and Social Adjustment Scale; TSQ, Traumatic Screening Questionnaire.

### Sample demographics and characteristics

As displayed in Fig. 1, 239 HSCWs self-referred to the pathway between April 2020 and April 2021. Table 1 provides the demographics of those who referred to the service (*n* = 239) and the final sample that underwent intervention (*n* = 156). The mean age of those who referred was 37.32 (s.d. = 10.59) years. The vast majority were female (84%). Approximately 61% of the sample was White and 33% reported that they were of a minority ethnic background; 6% did not report their ethnicity. The majority of participants belonged to allied health professionals (27%), admin and clerical (15%), and nursing and midwifery (31%) staff groups. Around a third (33%) were redeployed at the point of self-referring. Among those who were offered support (*n* = 159), these proportions of characteristics or demographics were statistically the same despite signposting to other services and drop-out. Table 1 provides *χ*^2^- and *t*-test *P*-values for demographic and time point 1 outcome measure comparisons between those who referred and those who underwent intervention, with no significant differences between the two groups found. Across all of the interventions offered (including those in combination), HSCWs attended 6.89 (s.d. = 5.37) sessions, on average.

### Symptom change

There was evidence that symptoms of depression, generalised anxiety and PTSD were lower post-intervention (time point 2), and that functioning was higher, compared with pre-intervention (time point 1) levels (Table 2). Although 156 were offered a form of support, not every HSCW provided scores at time point 2 because of drop-out or issues that resulted in missing data (e.g. practitioner or collection errors). Time point 2 completion rates differed by measure and ranged from 70% (WSAS) to 88% (PHQ-9). Baseline TSQ scores were only for those who reported a potentially traumatising incident and only those with a time point 1 TSQ score completed it at time point 2 (*n* = 42).

**Table 2:** Paired sample *t*-test results and mean symptom scores at time points 1 and 2 for those who completed intervention(s)

Measure	Time point 1 mean (s.d.)	Time point 2 mean (s.d.)	*t*-value	*d*-value	*P*-value
PHQ-9, *n* = 123	12.62 (5.16)	5.63 (3.63)	14.75	1.33	**<0.001**
GAD-7, *n* = 122	12.04 (5.02)	5.30 (3.60)	15.15	1.37	**<0.001**
WSAS, *n* = 110	17.50 (7.90)	9.50 (6.29)	9.79	0.93	**<0.001**
TSQ, *n* = 42	4.62 (2.91)	2.57 (2.57)	5.58	0.86	**<0.001**

*P* values in boldface are statistically significant (*P* = 0.05). PHQ-9, Patient Health Questionnaire-9; GAD-7, Generalised Anxiety Disorder-7; WSAS, Work and Social Adjustment Scale; TSQ, Traumatic Screening Questionnaire.

### GRI scores

Sixty-eight HSCWs (73.1%) stated that in regards to their mood and feelings, they felt ‘A lot better’, 23 (24.7%) felt ‘A little better’ and two (2.2%) felt ‘About the same’, compared with when they self-referred. The mean GRI score was 4.71, indicating large self-perceived improvements on average.

### Moderators of outcome measure changes

There was no evidence that ethnicity, staff group, redeployment status or intervention type(s) were associated with differences in symptom change on any of the measures. Ethnicity was not associated with PHQ-9 (*P* = 0.69), GAD-7 (*P* = 0.95) or WSAS (*P* = 0.33) score improvements. Staff group was not associated with PHQ-9 (*P* = 0.32), GAD-7 (*P* = 0.19) or WSAS (*P* = 0.98) score improvements. Redeployment status was not associated with PHQ-9 (*P* = 0.28), GAD-7 (*P* = 0.75) or WSAS (*P* = 0.08) score improvements. Intervention type(s) was not associated with PHQ (*P* = 0.90), GAD-7 (*P* = 0.46) or WSAS score improvements (*P* = 0.59).

### Symptom change by intervention

Post-intervention outcomes on each of the measures were not associated with intervention type(s), yet within each intervention type, there was evidence of reduced symptom scores post-intervention (Table 3).

**Table 3:** Mean scores for measures at time points 1 and 2, by intervention(s)

Intervention(s)/measure	Time point 1 mean (s.d.)	Time point 2 mean (s.d.)	Mean difference *P*-values
PFA			
PHQ-9, *n* = 48	10.92 (4.88)	5.08 (5.57)	<0.001
GAD-7, *n* = 48	10.52 (5.12)	4.98 (2.42)	<0.001
WSAS, *n* = 42	15.45 (7.70)	9.29 (6.32)	<0.001
TSQ, *n* = 22	4.41 (2.70)	1.60 (1.76)	<0.001
PFA + LICBT			
PHQ-9, *n* = 17	12.94 (5.44)	5.35 (2.67)	<0.001
GAD-7, *n* = 17	12.41 (5.98)	4.41 (2.98)	<0.001
WSAS, *n* = 13	14.38 (6.20)	9.69 (5.88)	0.018
TSQ, *n* = 7	4.00 (0.82)	2.14 (1.68)	0.011
PFA + HIT			
PHQ-9, *n* = 27	13.63 (5.01)	5.96 (3.71)	<0.001
GAD-7, *n* = 27	12.70 (3.83)	5.44 (3.38)	<0.001
WSAS, *n* = 23	19.44 (7.50)	10.67 (6.80)	<0.001
TSQ, *n* = 12	5.42 (4.03)	4.42 (3.29)	0.118
LICBT			
PHQ-9, *n* = 14	15.43 (5.87)	5.79 (3.53)	<0.001
GAD-7, *n* = 14	12.93 (5.34)	5.43 (4.52)	<0.001
WSAS, *n* = 12	20.25 (7.67)	7.83 (4.95)	<0.001
HIT			
PHQ-9, *n* = 16	12.91 (3.94)	6.25 (4.03)	<0.001
GAD-7, *n* = 16	14.31 (4.22)	6.81 (4.21)	<0.001
WSAS, *n* = 16	20.06 (8.90)	9.19 (6.86)	<0.001

TSQ scores were not collected or compared for LICBT and HIT cases at either time point because PFA did not form part of their support. *P*-values are provided for the time point 1–2 measure mean differences. PFA, psychological first aid; PHQ-9, Patient Health Questionnaire-9; GAD-7, Generalised Anxiety Disorder-7; WSAS, Work and Social Adjustment Scale; TSQ, Traumatic Screening Questionnaire; LICBT, low-intensity cognitive–behavioural therapy; HIT, high-intensity cognitive–behavioural therapy.

### Feedback

**Fig. 2:**
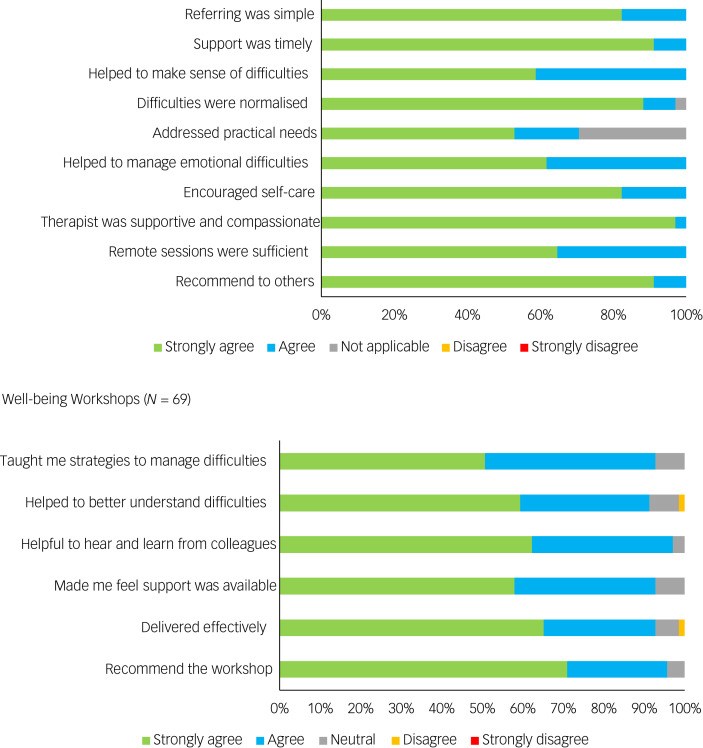
Feedback questionnaire results for psychological first aid sessions and well-being workshops.

Feedback was provided by 34 HSCWs (31%) who attended sessions of PFA. Feedback was largely positive (Fig. 2). The following quotes are taken from an open-ended item of the feedback form and highlight some aspects of PFA that they found beneficial:

‘Just talking and being able to share how I was feeling was really helpful, allowing me to open up. I also realised I wasn't the only one and that the problems I had were normal given the circumstances of the pandemic.’

‘I am someone who always puts others before myself, so it was great to have someone to support me in putting myself first for once – I need to look after myself with self-care.’

‘I feel like I have more coping strategies now, and as a result I am much better equipped to cope with the stress of my day-to-day life during the pandemic.’

Sixty-nine of the approximately 300 HSCWs that attended the well-being workshops completed the feedback questionnaire. Again, feedback was largely positive (Fig. 2). The following are example quotes taken from feedback forms of those who attended the well-being workshops:

‘Before, it was hard to have these conversations, as check-ins can often feel superficial, or we don't want to share our worries with colleagues. Creating a safe space such as this seems useful, and something we will try to implement as a team.’

‘All my feelings are valid and others feel the same - ‘I am not alone’. The biggest take home is to follow the plan, not the mood. I can be paralysed into inaction but I think this strategy makes a lot of sense and I am going to give it a try.’

‘It showed that as a group we need to be more open to how we feel and drop the ‘I can take it’ attitude and learn to be more open, honest and reflective. Definitely a good start and something I hope will be more regular to help change the medic mindset/culture.’

## Discussion

This study is the first quantitative evaluation of the effectiveness and acceptability of a psychological support pathway for HSCWs during COVID-19 in the UK, delivered by a primary care psychology service. Taken together, the one-to-one and well-being group support was offered to around 10%–15% of the acute physical healthcare trust (around 3658 employees) at the point of data analysis.^38^ In line with the aims of the evaluation, there was evidence of improved mental health and functioning post-intervention whether delivered alone (PFA, LICBT, HIT) or in combination (PFA and LICBT, PFA and HIT). No differences in effectiveness were observed based on ethnicity, redeployment status or staff grouping. Those HSCWs that gave feedback had very positive opinions of the PFA sessions and well-being workshops.

The evidence base for one-to-one interventions routinely offered in IAPT settings (LICBT and HIT) is well established, whereby around 50% of those with depression and anxiety disorders ‘recover’ as defined by the clinical thresholds of the PHQ-9 and GAD-7.^39^ This was mirrored by the depression and anxiety symptom change among HSCWs who received either LICBT or HIT, whereby both depression and anxiety scores moved from the ‘moderately severe’ range to the ‘mild’ or sub-clinical range, on average.^32,33^ Likewise, those who had either LICBT or HIT moved from the ‘moderately severe’ (>20) to the subclinical threshold (<10) of functional impairment.^34^ These outcomes were obtained within around the same number of sessions of LICBT or HIT provided in IAPT settings for non-HSCW populations.^3^

Although commonly used to mitigate the impact of crises, including disease outbreaks, on the mental health of individuals and communities, PFA does not have an established evidence base.^20^ This is largely because of an interaction between the unpredictable nature of crises, which require a rapid response, and the time-limiting procedures of obtaining ethics for research. Furthermore, when research has been possible, it has been very low quality.^21^ Therefore, a strength of the evaluation was the prompt collection of data, permitted by the routine data collection processes of IAPT services and the speed at which the pathway protocol was planned and implemented.^12^ Because of this, the paper has been able to report effectiveness findings for the use of PFA across various waves of COVID-19 infection that have defined the pandemic in the UK. These findings suggest that PFA may have a part to play in the reduction of depression and anxiety symptoms, as well as functional impairment, during and briefly after potentially traumatising incidents experienced by HSCWs in hospital or community settings. Although validation of this claim is outside the scope of the current paper, it is possible that PFA mitigates the need for further intervention, such as LICBT or HIT, by limiting the emergence of mental health problems. A reduction in trauma symptoms was also observed among HSCWs who had PFA, but surprisingly, the TSQ scores at baseline were low and only marginally above the threshold (≥5) that warrants further PTSD assessment.^36^ Despite concerns expressed early on in the pandemic, it is possible that HSCWs exhibit high levels of personal resilience in the face of traumatic events, perhaps as a result of their training.^6,40^ However, it is also plausible that some HSCWs underreport their symptoms because of barriers such as stigma. These improvements in symptoms cannot be solely attributed to PFA until large-scale controlled studies are conducted that build on naturalistic studies of small sample sizes, such as Kameno et al.^41^ Nevertheless, the results of the PFA feedback questionnaire supports the premise that the mechanisms of change instilled by the intervention, as summarised by Hobfoll et al,^24^ are perceived as helpful and acceptable by HSCWs.

The results of the evaluation also demonstrated the feasibility of offering PFA as a step 1 intervention in combination with LICBT or HIT, either as a prelude to formal therapy (steps 2 and 3) or in the midst of it, when the rising pressures of the pandemic disrupted its protocolised delivery. HSCWs who received sessions of both in this way also experienced improvements in depression, anxiety and functional impairment. Although further research is required to disentangle the interacting benefits of PFA and LICBT or HIT, it appears that practitioners were able to utilise PFA flexibly and in a way that bridges the gap between step 1 and steps 2 or 3 of the stepped-care model, while complementing the healing mechanisms of psychological therapies. In line with this, a recent systematic review reported that practitioners trained in PFA felt equipped with adequate skills and knowledge to support individuals in acute distress.^22^ This is especially important given the diverse needs of HSCWs, which can vary on the basis of the diverse layers and intersects of societal identity that comprise the individual, which might be accompanied by barriers to help-seeking.^42^ Although more research is needed, it has also been indicated that staff trained in PFA have lower levels of psychological distress when faced with COVID-19-related stressors.^43^ The experiences of practitioners delivering PFA as part of the evaluated service pathway have been explored and will be reported in a separate qualitative study.

There are several limitations of the above findings. First, follow-up data was not included in the evaluation, and therefore, it is unclear whether the observed improvements were maintained beyond the intervention(s). Causal associations could not be studied as there was no untreated or usual care control group, and it is likely that a number of those who reported benefits post-intervention spontaneously recovered.^44^ This is especially true of those who experienced a reduction in symptoms after sessions of PFA only, since evaluations of its use (including this study) have been unable to ascertain the proportion of outcomes that can be attributed to natural recovery over and above the intervention itself. This is because there was no opportunity for randomisation to the interventions, so confounding effects cannot be ruled out. In particular, we had no measure of pre-existing mental health and alternative experiences of care, which are associated with treatment prognosis irrespective of the type of treatment received.^45,46^ In addition, our ability to investigate sociodemographic moderators was limited to those data routinely collected in IAPT services. Others, such as social support, life events, relationship status and socioeconomic factors, may have affected the associations observed here. The use of naturalistic settings to provide the interventions and recruit participants could enhance generalisability, but selection biases owing to organisational and systemic factors unique to the Trust cannot be ruled out. Subsequently, there is a need for evidence-based guidelines for PFA, especially since its delivery can assume various forms subject to the context and culture it is delivered in.^21,24^ Likewise, more evaluations of PFA in other contexts are crucial.^47^ Finally, the uptake of PFA among HSCWs was relatively low compared with other studies, and those belonging to the medical and dental staff group were particularly underrepresented.^48^ The reasons for this are unclear, but it may be related to the opportunity afforded to HSCWs of the Trust to receive alternative interventions or because of barriers to access such as stigma, which may affect HSCWs of particular staff groups over others. This warrants future research.

Taken together, the findings of the current paper highlight the potential effectiveness and acceptability of a highly pragmatic psychological support pathway for HSCWs during COVID-19, utilising stepped- and phased-care principles. To build on this, further PFA-related research is warranted and, according to a recent systematic review, good-quality studies that ‘include randomisation, control groups, long-term follow-up periods, and sophisticated analytic designs and methods’ should be prioritised. Furthermore, studies should investigate the mechanisms of change linked to the intervention, establish standardised outcome measures and identify process performance indicators for PFA interventions.^49^ Finally, the experiences and well-being of mental health professionals offering support to HSCWs should be considered in line with the question ‘who helps the helpers?’, given the potential for vicarious trauma.^50^

## Data Availability

The data that support the findings of this study are available on request from the corresponding author, C.L.C. The data are not publicly available due to containing information that could compromise the privacy of research participants; for example, demographic data, job role and staff grouping. Because HHFT is a small organisation, the data could be triangulated to potentially identify employees who access confidential psychological services.

## Supplementary material

For supplementary material accompanying this paper visit http://doi.org/10.1192/bjo.2023.66. click here to view supplementary material
