# Supplementary material for: A service evaluation of phased- and stepped-care psychological support for health and social care workers during the COVID-19 pandemic
Source: BJPsych Open. 2023 May 25;9(3):e95. doi: 10.1192/bjo.2023.66 (PMC10228210; doi:10.1192/bjo.2023.66)

**Supplementary Figure 1**

*A Bar Chart to Represent Mean Symptom Change across all Intervention Types and Combinations*


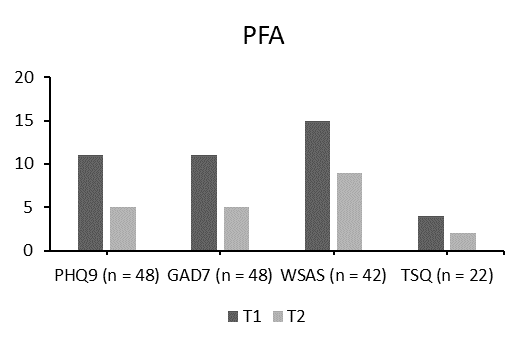

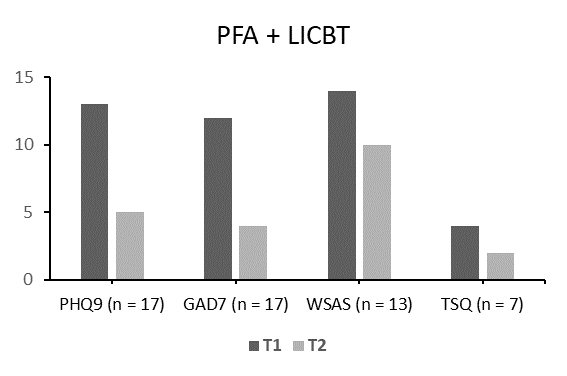

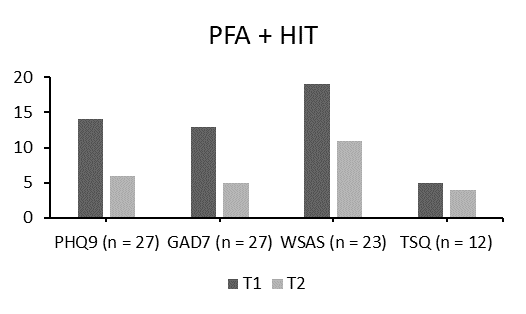

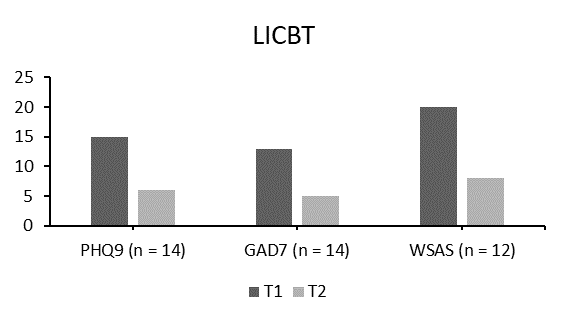

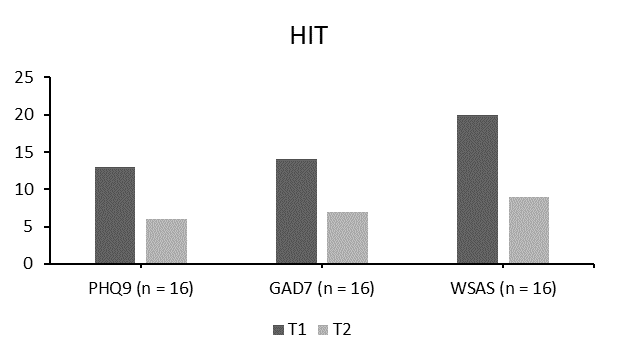

Supplement: Supplementary file 1 [file bjosup.zip › S2056472423000662sup001.docx]
