# Supplementary material for: A service evaluation of phased- and stepped-care psychological support for health and social care workers during the COVID-19 pandemic
Source: BJPsych Open. 2023 May 25;9(3):e95. doi: 10.1192/bjo.2023.66 (PMC10228210; doi:10.1192/bjo.2023.66)
Supplement: Supplementary file 1 [file bjosup.zip › S2056472423000662sup002.docx]

**Supplementary Material – Feedback Questionnaires**

**Psychological First Aid (PFA) Feedback Questionnaire**

Below are a series of questions, please select the option that best applies to your experience or the response that reflects the extent you agree with the statement

**How did you hear about our service?** *Briefing emails colleague manager / supervisor website*

other:

|  | **Strongly Disagree** | **Disagree** | **Neither Agree or Disagree** | **Agree** | **Strongly Agree** |
| --- | --- | --- | --- | --- | --- |
| *Referring to the service was straightforward* |  |  |  |  |  |
| *I was offered support in a timely manner* |  |  |  |  |  |
| *The sessions helped me to make sense of or understand my difficulties better* |  |  |  |  |  |
| *My feelings were made to seem understandable given my recent experiences (i.e. normalized)* |  |  |  |  |  |
| *I was supported to think of ways to get my practical needs at work met – e.g. PPE (*includes employment support*)* |  |  |  |  |  |
| *I was supported to think of ways I could cope with my emotional difficulties* |  |  |  |  |  |
| *Self-care* (i.e. looking after myself*) was encouraged in my sessions* |  |  |  |  |  |
| *My therapist was supportive and understanding* |  |  |  |  |  |
| *The sessions remained helpful over the phone or Attend Anywhere* |  |  |  |  |  |
| *I would recommend the service to others* |  |  |  |  |  |

What barriers were there to accessing the service?

What aspects of your experience, if any, would you change?

What did you take away from the sessions?

**Wellbeing Workshop Feedback Questionnaire**

Below are a series of questions, please select the option that best applies to your experience or the response that reflects the extent you agree with the statement

|  | **Strongly Disagree** | **Disagree** | **Neither Agree or Disagree** | **Agree** | **Strongly Agree** |
| --- | --- | --- | --- | --- | --- |
| *"The workshop taught me strategies to manage common difficulties"* |  |  |  |  |  |
| *"The workshop helped me to better understand some of the difficulties we face as healthcare staff"* |  |  |  |  |  |
| *"It was helpful to hear and learn about my colleagues experiences"* |  |  |  |  |  |
| *"The workshop made me feel that support was available, if needed"* |  |  |  |  |  |
| *"The workshop was delivered effectively"* |  |  |  |  |  |
| *"I would recommend the workshop to other teams"* |  |  |  |  |  |

What will you take away from the workshop?
